# Supplementary material for: Government responses and COVID-19 deaths: Global evidence across multiple pandemic waves
Source: PLoS One. 2021 Jul 9;16(7):e0253116. doi: 10.1371/journal.pone.0253116 (PMC8270409; doi:10.1371/journal.pone.0253116)
Supplement: S1 File — (DOCX) [file pone.0253116.s001.docx]

**S1 File**

***Government responses and COVID-19 deaths: global evidence across multiple pandemic waves.***

Thomas Hale^1*^ , Noam Angrist^1,^ Andrew J. Hale^2^, Beatriz Kira^1^, Saptarshi Majumdar^1^, Anna Petherick^1^, Toby Phillips^1^, Devi Sridhar^3^, Robin N. Thompson^4^, Samuel Webster^5^**,** Yuxi Zhang^1^

*^1^* Blavatnik School of Government, University of Oxford, Oxford, United Kingdom

**^2^**Larner College of Medicine at the University of Vermont, Burlington, Vermont, United States

^3^Professor, University of Edinburgh, Edinburgh, Scotland, United Kingdom

^4^ Mathematics Institute, University of Warwick, UK

^5^Unaffiliated, London, United Kingdom

*Corresponding author: Thomas.hale@bsg.ox.ac.uk

S1 Table: Coding instrument for indicators of government responses

S2.1-S2.4 Tables: Robustness checks

List of data collectors

S1 Fig: Waves by country

List of countries included in the analysis.

**S1 Table. Coding instrument for indicators of government responses**

| **ID** | **Name** | **Description** | **Measurement** | **Coding** |
| --- | --- | --- | --- | --- |
| C1 | C1_School closing | Record closings of schools and universities | Ordinal scale | 0 - no measures 1 - recommend closing 2 - require closing (only some levels or categories, eg just high school, or just public schools) 3 - require closing all levels Blank - no data |
|  | C1_Flag |  | Binary flag for geographic scope | 0 - targeted 1- general Blank - no data |
| C2 | C2_Workplace closing | Record closings of workplaces | Ordinal scale | 0 - no measures 1 - recommend closing (or recommend work from home) 2 - require closing (or work from home) for some sectors or categories of workers 3 - require closing (or work from home) for all-but-essential workplaces (eg grocery stores, doctors) Blank - no data |
|  | C2_Flag |  | Binary flag for geographic scope | 0 - targeted 1- general Blank - no data |
| C3 | C3_Cancel public events | Record cancelling public events | Ordinal scale | 0 - no measures 1 - recommend cancelling 2 - require cancelling Blank - no data |
|  | C3_Flag |  | Binary flag for geographic scope | 0 - targeted 1- general Blank - no data |
| C4 | C4_Restrictions on gatherings | Record limits on private gatherings | Ordinal scale | 0 - no restrictions 1 - restrictions on very large gatherings (the limit is above 1000 people) 2 - restrictions on gatherings between 101-1000 people 3 - restrictions on gatherings between 11-100 people 4 - restrictions on gatherings of 10 people or less Blank - no data |
|  | C4_Flag |  | Binary flag for geographic scope | 0 - targeted 1- general Blank - no data |
| C5 | C5_Close public transport | Record closing of public transport | Ordinal scale | 0 - no measures 1 - recommend closing (or significantly reduce volume/route/means of transport available) 2 - require closing (or prohibit most citizens from using it) Blank - no data |
|  | C5_Flag |  | Binary flag for geographic scope | 0 - targeted 1- general Blank - no data |
| C6 | C6_Stay at home requirements | Record orders to "shelter-in-place" and otherwise confine to the home | Ordinal scale | 0 - no measures 1 - recommend not leaving house 2 - require not leaving house with exceptions for daily exercise, grocery shopping, and 'essential' trips 3 - require not leaving house with minimal exceptions (eg allowed to leave once a week, or only one person can leave at a time, etc) Blank - no data |
|  | C6_Flag |  | Binary flag for geographic scope | 0 - targeted 1- general Blank - no data |
| C7 | C7_Restrictions on internal movement | Record restrictions on internal movement between cities/regions | Ordinal scale | 0 - no measures 1 - recommend not to travel between regions/cities 2 - internal movement restrictions in place Blank - no data |
|  | C7_Flag |  | Binary flag for geographic scope | 0 - targeted 1- general Blank - no data |
| C8 | C8_International travel controls | Record restrictions on international travel  Note: this records policy for foreign travellers, not citizens | Ordinal scale | 0 - no restrictions 1 - screening arrivals 2 - quarantine arrivals from some or all regions 3 - ban arrivals from some regions 4 - ban on all regions or total border closure Blank - no data |
| H1 | H1_Public information campaigns | Record presence of public info campaigns | Ordinal scale | 0 - no Covid-19 public information campaign 1 - public officials urging caution about Covid-19 2- coordinated public information campaign (eg across traditional and social media) Blank - no data |
|  | H1_Flag |  | Binary flag for geographic scope | 0 - targeted 1- general Blank - no data |
| The 2 indicators below (H2 and H3) are not included in the Stringency Index | | | | |
| H2 | H2_Testing policy | Record government policy on who has access to testing  Note: this records policies about testing for current infection (PCR tests) not testing for immunity (antibody test) | Ordinal scale | 0 - no testing policy 1 - only those who both (a) have symptoms AND (b) meet specific criteria (eg key workers, admitted to hospital, came into contact with a known case, returned from overseas) 2 - testing of anyone showing Covid-19 symptoms 3 - open public testing (eg "drive through" testing available to asymptomatic people) Blank - no data |
| H3 | H3_Contact tracing | Record government policy on contact tracing after a positive diagnosis  Note: we are looking for policies that would identify all people potentially exposed to Covid-19; voluntary bluetooth apps are unlikely to achieve this | Ordinal scale | 0 - no contact tracing 1 - limited contact tracing; not done for all cases 2 - comprehensive contact tracing; done for all identified cases |

**S2: Robustness checks**

**S2.1 Table:** Association of Government Response Stringency and Deaths by Wave controlling for country specific covariates (GDP, hospital bed availability and comorbidities)

|  | (1) | (2) | (3) | (4) |
| --- | --- | --- | --- | --- |
|  | Pooled Estimates for All Countries | One-Wave Countries | Two-Wave Countries | Three-Wave Countries |
| Lagged Stringency by 28 Days | -0.009*** |  |  |  |
|  | (0.001) |  |  |  |
|  | [0.000] |  |  |  |
| Lagged Stringency by 28 Days: Wave 1 |  | -0.004*** | -0.003*** | -0.020*** |
|  |  | (0.001) | (0.001) | (0.002) |
|  |  | [0.000] | [0.000] | [0.000] |
| Lagged Stringency by 28 Days: Wave 2 |  |  | -0.007*** | -0.031*** |
|  |  |  | (0.001) | (0.002) |
|  |  |  | [0.000] | [0.000] |
| Lagged Stringency by 28 Days: Wave 3 |  |  |  | -0.028*** |
|  |  |  |  | (0.003) |
|  |  |  |  | [0.000] |
| Countries | 113 | 40 | 63 | 10 |
| R2 | 0.69 | 0.69 | 0.71 | 0.81 |
| Country Controls | Yes | Yes | Yes | Yes |
| Time Trend | Yes | Yes | Yes | Yes |
| Lagged Deaths Control | Yes | Yes | Yes | Yes |

*Notes*: All regressions coefficients are included in the table, followed by standard errors in parentheses and p-values in square brackets. Stars signify statistical significance at conventional thresholds.

**S2.2 Table:** Association of Government Response Index and Deaths by Wave

|  | (1) | (2) | (3) | (4) |
| --- | --- | --- | --- | --- |
|  | Pooled Estimates for All Countries | One-Wave Countries | Two-Wave Countries | Three-Wave Countries |
| Lagged Government Response by 28 Days | -0.012*** |  |  |  |
|  | (0.001) |  |  |  |
|  | [0.000] |  |  |  |
| Lagged Government Response by 28 Days: Wave 1 |  | -0.007*** | -0.010*** | -0.046*** |
|  |  | (0.002) | (0.001) | (0.003) |
|  |  | [0.000] | [0.000] | [0.000] |
| Lagged Government Response by 28 Days: Wave 2 |  |  | -0.011*** | -0.058*** |
|  |  |  | (0.001) | (0.003) |
|  |  |  | [0.000] | [0.000] |
| Lagged Government Response by 28 Days: Wave 3 |  |  |  | -0.051*** |
|  |  |  |  | (0.003) |
|  |  |  |  | [0.000] |
| Countries | 113 | 40 | 63 | 10 |
| R2 | 0.76 | 0.75 | 0.76 | 0.82 |
| Country Fixed Effects | Yes | Yes | Yes | Yes |
| Time Trend | Yes | Yes | Yes | Yes |
| Lagged Deaths Control | Yes | Yes | Yes | Yes |

*Notes*: All regressions coefficients are included in the table, followed by standard errors in parentheses and p-values in square brackets. Stars signify statistical significance at conventional thresholds.

**S2.3 Table:** Association of Subnational Policy Response (State wise Stringency Index in USA and Brazil) and Deaths by Wave

|  | (1) | (2) | (3) | (4) |
| --- | --- | --- | --- | --- |
|  | Pooled Estimates for All Countries | One-Wave Countries | Two-Wave Countries | Three-Wave Countries |
| Lagged Stringency by 28 Days | -0.005*** |  |  |  |
|  | (0.001) |  |  |  |
|  | [0.000] |  |  |  |
| Lagged Stringency by 28 Days: Wave 1 |  | -0.006*** | -0.003*** | -0.014*** |
|  |  | (0.001) | (0.001) | (0.002) |
|  |  | [0.000] | [0.000] | [0.000] |
| Lagged Stringency by 28 Days: Wave 2 |  |  | -0.006*** | -0.017*** |
|  |  |  | (0.001) | (0.002) |
|  |  |  | [0.000] | [0.000] |
| Lagged Stringency by 28 Days: Wave 3 |  |  |  | -0.016*** |
|  |  |  |  | (0.002) |
|  |  |  |  | [0.000] |
| Countries | 149 | 41 | 88 | 21 |
| R2 | 0.72 | 0.74 | 0.72 | 0.70 |
| Country and Subnational Fixed Effects | Yes | Yes | Yes | Yes |
| Time Trend | Yes | Yes | Yes | Yes |
| Lagged Deaths Control | Yes | Yes | Yes | Yes |

*Notes*: All regressions coefficients are included in the table, followed by standard errors in parentheses and p-values in square brackets. Stars signify statistical significance at conventional thresholds.

**S2.4 Table:** Association of Government Response Stringency and Deaths by Wave controlling for countries’ testing and contact tracing responses.

|  | | | | (1) | | | (2) | | (3) | (4) |
| --- | --- | --- | --- | --- | --- | --- | --- | --- | --- | --- |
|  | | | | Pooled Estimates for All Countries | | | One-Wave Countries | | Two-Wave Countries | Three-Wave Countries |
| Lagged Stringency by 28 Days | | | | -0.006*** | | | | |  |  |
|  | | | | (-0.001) | | |  | |  |  |
|  | | | | [0.000] | | |  | |  |  |
| Lagged Stringency by 28 Days: Wave 1 | | |  | | | | -0.006*** | | -0.004*** | -0.019*** |
|  | | | |  | | | (-0.001) | | (-0.001) | (-0.002) |
|  | | | |  | | | [0.000] | | [0.000] | [0.000] |
| Lagged Stringency by 28 Days: Wave 2 | |  | | | |  | | | -0.008*** | -0.028*** |
|  | | | |  | | |  | | (-0.001) | (-0.002) |
|  | | | |  | | |  | | [0.000] | [0.000] |
| Lagged Stringency by 28 Days: Wave 3 |  | | | |  | | |  | | -0.024*** |
|  | | | |  | | |  | |  | (-0.003) |
|  | | | |  | | |  | |  | [0.000] |
| Countries | | | | 113 | | | 40 | | 63 | 10 |
| R2 | | | | 0.76 | | | 0.75 | | 0.76 | 0.81 |
| Country Fixed Effects | | | | Yes | | | Yes | | Yes | Yes |
| Time Trend | | | | Yes | | | Yes | | Yes | Yes |
| Lagged Deaths Control | | | | Yes | | | Yes | | Yes | Yes |
| Testing and Contact Tracing Controls | | | | Yes | | | Yes | | Yes | Yes |

*Notes*: All regressions coefficients are included in the table, followed by standard errors in parentheses and p-values in square brackets. Stars signify statistical significance at conventional thresholds.

**List of data collectors**

Adil Sayeed
 Aditya Lolla
 Adrian Wang Xinting
 Ahmed Safar
 Aidana Arynbek
 Alejandrina Cripovich
 Alexsander Silva Farias
 Alfredo Ortega
 Ali Arsalan Pasha Siddiqui
 Alice Eddershaw
 Alice Graham
 Alice Secheresse
 Alice Vodden
 Aline Tognini
 Allen Haugh
 Alonso Moran de Romana
 Ana Lucia Villagran
 Anandam Sarcar
 André Parente Houang
 Andrea Garaiova
 Andrea Klaric
 Andreea Anastasiu
 Andrew Brown
 Andrew Iupati
 Andrew Read
 Andrew Wood
 Andrey Krachkov
 Anindita K. Listya
 Anita Pant
 Anjali Viswamohanan
 Ann Hagen
 Anna Bruvere
 Anna Paula Ferrari Matos
 Anna Petherick
 Anna Welsh
 Annalena Pott
 Annamarie Candler
 Anneloes Hoff
 Anthony Sudarmawan
 Anupah Makoond
 Ariana Detmar
 Ariq Hatibie
 Arkar Hein
 Arthur Lau
 Ayanna Griffith
 Babu Ahamed
 Bárbara Prado Simão
 Barbara Roggeveen
 Barbora Bacyte
 Beatriz Cristina Rodrigues SilvaDébora Nery Schwartz
 Beatriz Franco
 Beatriz Kira
 Beatriz Pioltine Macedo Costato
 Ben Luria
 Ben Weber
 Benjamin Ignac
 Benjamin Parker
 Benjamin Peart
 Bilal Majeed
 Bill McCluskey
 Blessing Oluwatosin Ajimoti
 Bolorerdene Battsengel
 Bpriya Lakshmy Tbalasubramaniam
 Bronwyn Gavine
 Bruno da Cunha de Oliveira
 Bruno Stucchi
 Bugei Nyaosi
 Camilla Sacchetto
 Carla Almeida da Vila
 Carolina Martinelli
 Carolina Scherer Beidacki
 Caroline Carthy
 Caroline Frassão
 Caroline Weglinski
 Cassy Inman
 Celso Antônio Coelho Júnior
 Chandra Sekhar Viswanadha
 Changyu (Erin) Yan
 Charlotte Newton
 Charlotte Rougier
 Chenxi Zhu
 Chloe Axford
 Chloë Mayoux
 Christian Lumley
 Christopher Yoannou
 Clara Pavillet
 Clara Portwood
 Clarissa Lim
 Clea Boorman
 Connor Lyons
 Cristhian Pulido
 Cynthia Waliaula
 Dan Fay
 Dan Grinevics
 Dan Mocanu
 Dane Alivarius
 Dang Dao Nguyen
 Daniel Pereira Cabral
 Daniela Ferraz Bertholini
 Daniela Mayerova
 Dar-Yin Li
 Dário Kuteev Moreira
 Davi Mamblona Marques Romão
 Davi Mancebo Fernandes
 David Johnson
 David McKinnon
 Dayane Ferreira
 Déborah Palacio do Sacramento
 Delgermaa Munkhgerel
 Delia Tegnalia
 Denilson Soares Gomes Junior
 Derek Messling
 Diane Brandt
 Diogo Mussalem Smethurst
 Dita Listya
 Dr Grace Mzumara
 Edmund Derby
 Edward O'Brien
 Ehsan Rafian
 Elaine Fung
 Eleanor Altamura
 Elena Terenzi
 Elisa Codonho Premazzi
 Elisabeth Mira Rothweiler
 Elisangela Oliveira de Freitas
 Ellen Sugrue
 Emily Cameron-Blake
 Emily Nguyen
 Emma Leonard
 Emmanuel Mawuli Abalo
 Eric Cheng
 Erin Kanzig
 Eshitha Vaz
 Ethan Teo
 Fabiana da Silva Pereira
 Farah Sayad
 Fatima Zehra Naqvi
 Felipe Dias Gonçalves
 Felipe Natil Martins Moreira
 Felipe Paiva Pinto
 Felipe Rodrigues Monteiro
 Femi Adebola
 Finn Klebe
 Fiona Ching Ming Hsu
 Francesca Basile
 Francesca Lovell-Read
 Francesca Valmorbida McSteen
 Francesco Moiraghi
 Frederic Michaelsen
 Gabriel de Azevedo Soyer
 Gabriel Dowuona
 Gabriel Podesta
 Gabriel Rossini
 Gabriella Henrique Targino Monteiro
 Gaia Lisi
 Garima Rana
 Gautam Dambekodi
 Gemma Mortell
 George Sheppard
 Giulia Biasibetti
 Giulia Pirolo
 Grace Mzumara
 Guilherme Macedo
 Guilherme Ramos
 Guillermo Miranda
 Gustavo Henrique Luz Silva
 Haiming Wu
 Haiyun Deng
 Hakeem Onasanya
 Hakim Ronaque
 Hala Sheikh Al Souk
 Hannah Klim
 Hao Zha
 Harikharan Krishnaraju
 Hatim Hussain
 Heather Walker
 Helen Tatlow
 Helene Jow
 Helórya Santiago de Souza
 Henrique Oliveira da Motta
 Henry Annan
 Hermann Fernandes Pais
 Hiu Hung Tse
 Horácio Figueira de Moura Neto
 Hui Zhou
 Huma Zile
 Hunter McGuire
 Hyerean Yoo
 Ifigenia Xifre Villar
 Ilya Zlotnikov
 Inaara Gulamhussen
 Inaara Sundargy
 India Clancy
 Ingrid Maria Johansen
 Isabel Jorgensen
 Isabel Seelaender Costa Rosa
 Isabela Blumm
 Isabella Borges Ávila
 Isabelye dos Santos Mendes
 Israa Mohammed
 Iyone Agboraw
 Jai Wei
 Jaime Weber
 Jake Lerner
 James Fox
 James Green
 Jamie Weber
 Jason Larson
 Javier Pardo-Diaz
 Jay Harley
 Jeanna Kim
 Jenna Hand
 Jennifer Gunther
 Jennifer Lim
 Jeremy Ng
 Jes Shultz
 Jess Barreto
 Jessica Anania
 Jialin Xi
 Jianjun Wu
 Jiayi Deng
 Jiayi LI
 Jilin Zeng
 Jimmy Kwong
 Jinmeng Zhang
 Joanna Klimczak
 João Claudio Faria Machado
 João Ferreira da Silva
 João Gabriel de Paula Resende
 João Pires Mattar
 Johannes Dommnich
 John Miller
 Joohee Uhm
 Jorge Luis Revilla
 José Renato Venâncio Resende
 Joseph Ssentongo
 Joy Carveth
 Juan David Gutierrez
 Judy Cossins
 Judy Nguyen
 Juhi Kore
 Julia Sawatzky
 Juliana Moura Bueno
 Juliana Novaes
 Juliet O'Brien
 Ka Yu Wong
 Kaisa Saarinen
 Kaitlyn Green
 Kangning Zhang
 Karoline Becker
 Kasia Whitaker
 Katherine McCreery
 Katherine Tyson
 Katrina Marina
 Katy Aymar
 Kaushal Jain
 Kaushalya Gupta
 Kelly Daniels
 Kristie Jameson
 Kumar Shastry
 Kurt Sant
 Lam Quynh Vo
 Lama Khaiyat
 Lana Ahmad
 Larissa Cristina Margarido
 Laura Angelica Chavez-Varela
 Laura Chamberlain
 Laura de Lisle
 Laura dos Santos Boeira
 Laura Hallas
 Leana Diekmann
 Leanne Giordono
 Leire Gonzalez Yubero
 Leslie Fraser
 Letícia Barbosa Plaza
 Lia Stefanovich
 Lian Najami
 Liene Kaori Asahi Baptista
 Lilas Mercuriali
 Liliana Estrada Galindo
 Lin Shi
 Linrui Zhong
 Lione Alushula
 Liu Victoria Yang
 Liu Zhang
 Liviu Dimitriu
 Lorena G Barberia
 Louisa-Madeline Singer
 Lucia Soriano
 Lucy Ellen
 Lucy Goodfellow
 Luiz Eduardo Barbieri Bedendo
 Luiz Guilherme Roth Cantarelli
 Luyao Ren
 Manikarnika Dutta Dutta
 Manjit Nath
 Marcela Mello Zamudio
 Marcela Reynoso Jurado
 Marcelle Costa Marinho
 Marcelo Arruda Candido
 Marco Antonio Silva Costa
 Mareeha Kamran
 Maria Carolina Gachido
 Maria Clara Leme de Oliveira
 María de los Ángeles Lasa
 Maria Leticia Claro
 Maria Luciano
 Maria Paz Astigarraga Baez
 Maria Puolakkainen
 Mariam Raheem
 Mariana Costa Oliveira Morais
 Mariana Victoria Braga Resende
 Marianne Lafuma
 Marie Mavrikios
 Marília Camargo Miyashiro
 Mark Deakin
 Marryam Ishaq
 Marta Koch
 Martha Stolze
 Martina Lejtreger
 Marwa Ghoname
 Maryann Heil
 Matheus Mariano
 Matheus Porto Lucena
 Matheus Ricardo Gonçalves Barbosa
 Matilde Stronell
 Maurice Kirschbaum
 Maurício Nardi Valle
 Maxime Bourdier
 Megan McDowell
 Melissa Toh
 Melody Leong
 Meskerem Aleka Kebede
 Michael Chen
 Michelle Chan
 Michelle Sharma
 Mikafui Dzotsi
 Mildred Aziengbe
 Minah Rashad
 Minsoo Bae
 Miriam Pittalis
 Monika Pyarali
 Morgan Grobin
 Moza Ackroyd
 Muktai Panchal
 Myank Mawar
 Nadia Nasreddin
 Nadine Dogbe
 Natalia Brigagão
 Natália Colvero Maraschin
 Natália de Paula Moreira
 Natalia Elizabeth Espinola Lopez
 Nate Dolton-Thornton
 Nathan Felipe Caetano da Silva
 Nathaniel Dolton-Thornton
 Natsuno Shinagawa
 Negin Shahiar
 Nicholas Wan
 Nicole Guedes Barros
 Nicole Gump
 Nicole Nanci
 Nikhil Tekwani
 Noam Angrist
 Nomondalai Batjargal
 Oksana Matiiash
 Olga Romanova
 Olivia Route
 Pamela Gongora Salazar
 Paola Del Carpio Ponce
 Paola Schietekat Sedas
 Paraskevas Christodoulopoulos
 Patricia Silva Castillo
 Paul Anderson
 Paul Lawson
 Pedro Arcain Riccetto
 Pedro Santana Schmalz
 Pollyana Pacheco Lima
 Prabhakar Chandramouli
 Prakrit Prasad
 Praveen Rajendran
 Precious Oluwadara Olajide
 Prianka Rao
 Primrose Adjepong
 Priya Lakshmy Tbalasubramaniam
 Priyanka Bijlani
 Purna Chandra Panda
 Qianyi Ye
 Qing Yang
 Qingling Kong
 Qiyuan Dong
 Quynh Lam Vo (Lam)
 Rachel Dixon
 Rachelle Koch
 Rafael Goldszmidt
 Rahima Hanifa
 Rancy Chadha
 Randy Taufik
 Raymond Pottebaum
 Rayssa Deps Bolelli
 Rene' Landers
 Rhona Rahmani
 Ricardo Miranda Rocha Leitao
 Richard Florance
 Richard James Chapler
 Jr.
 Robert Gorwa
 Roda Mohammed
 Rodrigo Furst de Freitas Accetta
 Rose Wachuka Macharia
 Rotimi Elisha Alao
 Rushay Naik
 Ruwa Mahdi
 Saba Mahmood
 Safa Khan
 Sagar Grewal
 Salim Salamah
 Sam Webster
 Samantha Harris
 Samson Leung
 Samuel Smith
 San Jameson
 Sandra Sajeev
 Saptarshi Majumdar
 Sara Sethia
 Sasidhar Gali
 Scott Latham
 Scott McCullers
 Sena Pradipta
 Serene Singh
 Seun B. Adebayo
 SeungCheol Ohk
 Seungeun Yi
 Shabana Basij-Rasikh
 Shane Fitzsimons
 Shannon Costello
 Shannon Murray
 Shannon Smith
 Shelly Lim
 Shirley Chen
 Shiwen Lai
 Shoaib Khan
 Shubo Zhang
 Silvia Shen
 Simon Powell
 Simphiwe Stewart
 Siqi Liu
 Siu Cheng
 Sophie Pearlman
 Soumaya Belaid
 Stefaan Sonck Thiebaut
 Stephanie Guyett
 Stephen Hayes
 Suganthan Asokan
 Swathi Rayasam
 Syed Shoaib Hasan Rizvi
 Sze Oh
 Sze Tung Lam
 Taís Pelinson Gomes da Silva
 Tamoi Fujii
 Tania Calle
 Tanyah Hameed
 Tatianna Mello Pereira da Silva
 Tatsuya Yasui
 Tebello Qhotsokoane
 Teresa Soter Henriques
 Terrence Epie
 Teruki Takiguchi
 Tetsekela Anyiam-Osigwe
 Theo Bernard
 Thomas Benson
 Thomas Birdseye
 Thomas Boby
 Thomás Castanheira Manfrinatti
 Thomas Rowland
 Thomas Stubbs
 Tilbe Atav
 Tim Nusser
 Tina Chim
 Tiphaine Le Corre
 Tiwa Ighomuaye
 Toby Phillips
 Tom Hale
 Trevor Edobor
 Twan van der Togt
 Ulla Mikkelsen
 Ulrike Gruber-Gremlich
 Ursula Panzner
 Ursule Demael
 Uttara Narayan
 Veronique Gauthier
 Victor Mtaki
 Victoria Cavero
 Vijay Krishna Palepu
 Vinícius Sanches Pontirolle
 Walter Vinicius Ribeiro Cancelieri
 Wei Sean Melvin Ting
 Will Marshall
 William Dowling
 William Hart
 Winni Yang
 Xema Pathak
 Xinrui Wang
 Yanying Lin
 Yaowen Deng
 Yexuan Zhu
 Yinqiu Zheng
 Yiwen Sun
 Yiwen Zhang
 Yizhou Pan
 Yulia Taranova
 Yuxi Zhang
 Yuxin Ma
 Zachary Parsons
 Zara Abdurahaman
 Zara Raheem
 Zelie Kasten
 Zile Huma
 Zilin Tu
 Ziqi Zhou
 Ziqing Huang
 Zoe Lin
 Zoha Imran
 Zoha Minal Imran
 Zongyue Liu
 Zunaira Mallick

**Examples of waves by country**

Single Wave


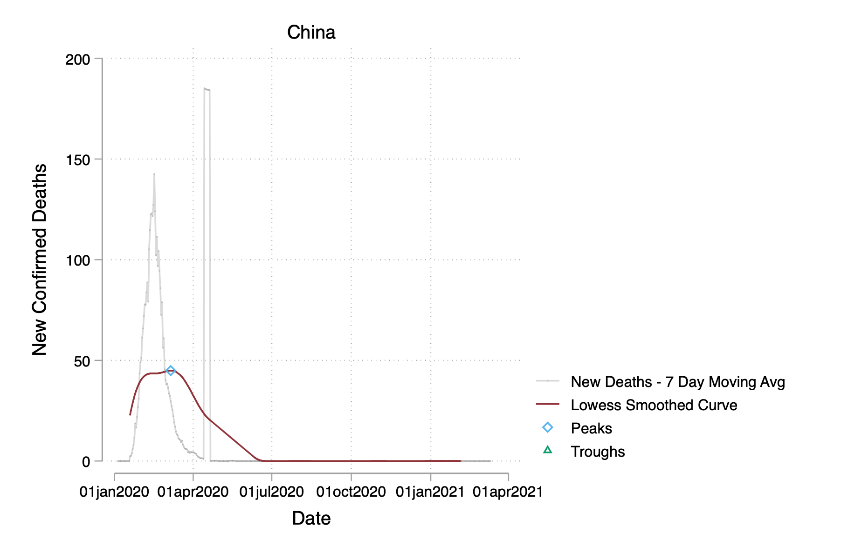


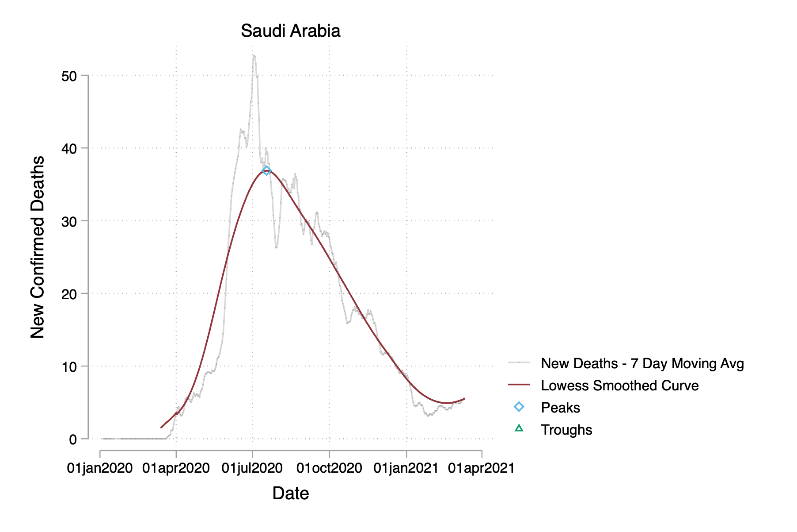


Two Waves


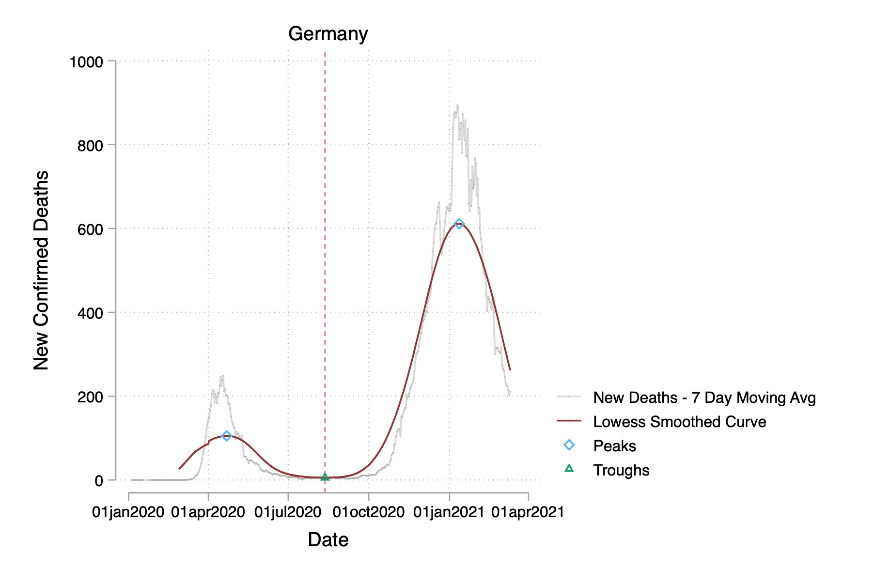


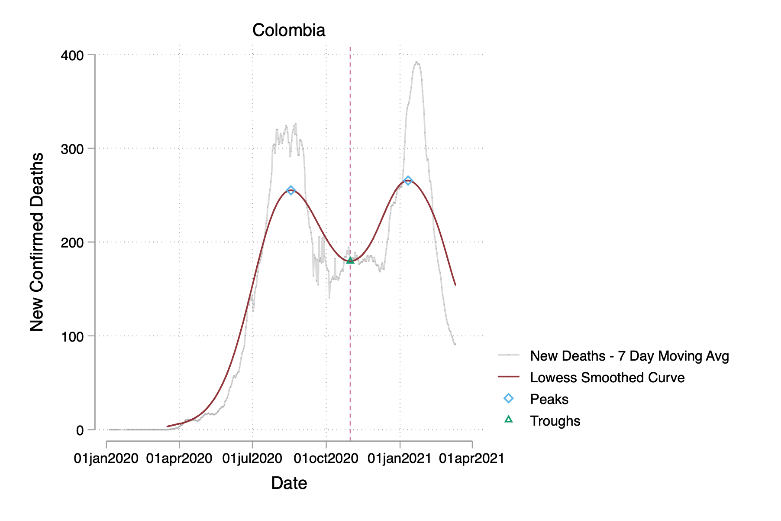


Three Waves


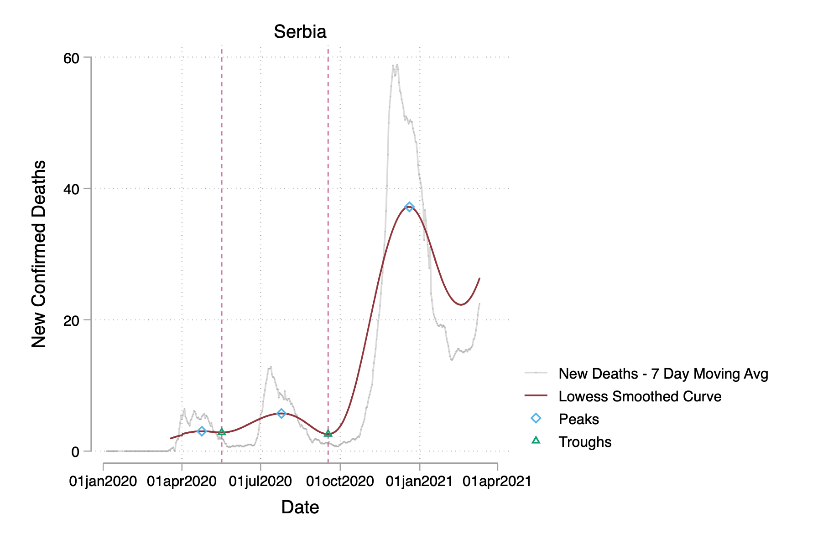

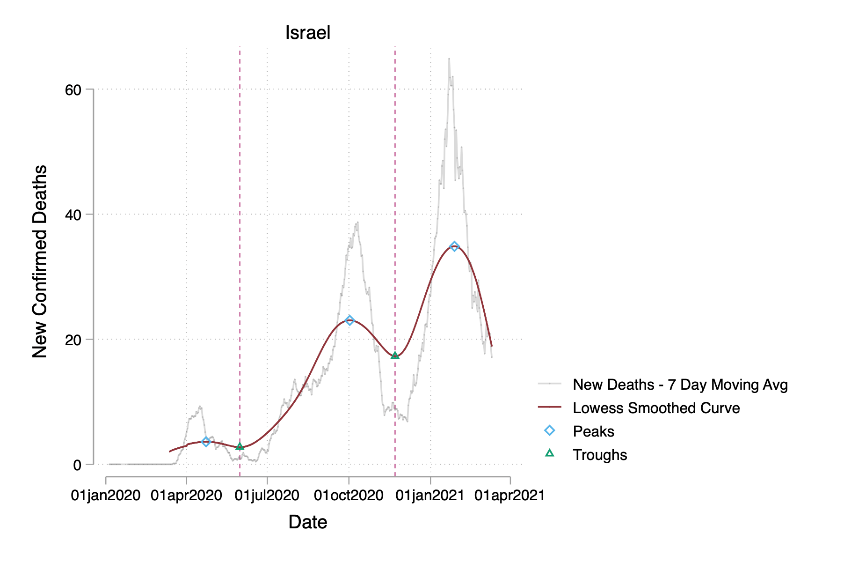


**Countries included in the analysis**

Albania, Angola, Argentina, Australia, Botswana, Bulgaria, China, Croatia, Democratic Republic of Congo, Eswatini, Finland, Georgia, Greece, India, Indonesia, Ireland, Latvia, Lebanon, Lithuania, Luxembourg, Madagascar, Malawi, Malaysia, Myanmar, Netherlands, Norway, Palestine, Saudi Arabia, Senegal, Slovak Republic, Slovenia, Somalia, Sri Lanka, Sudan, Syria, Tunisia, Uruguay, Uzbekistan, Yemen, Zimbabwe, Afghanistan, Austria, Azerbaijan, Bangladesh, Belarus, Bolivia, Bosnia and Herzegovina, Brazil, Canada, Chile, Colombia, Costa Rica, Cuba, Czech Republic, Denmark, Dominican Republic, Egypt, El Salvador, Estonia, Ethiopia, France, Germany, Ghana, Guatemala, Honduras, Hungary, Iran, Iraq, Italy, Jamaica, Jordan, Kazakhstan, Kenya, Kosovo, Kyrgyz Republic, Libya, Mexico, Moldova, Morocco, Mozambique, Namibia, Nepal, Nigeria, Oman, Pakistan, Panama, Paraguay, Peru, Philippines, Poland, Portugal, Puerto Rico, Romania, Russia, South Africa, South Korea, Sweden, Turkey, Ukraine, United Arab Emirates, United Kingdom, Venezuela, Zambia, Algeria, Bahrain, Belgium, Israel, Japan, Kuwait, Serbia, Spain, Switzerland, United States
