# Supplementary material for: Government responses and COVID-19 deaths: Global evidence across multiple pandemic waves
Source: PLoS One. 2021 Jul 9;16(7):e0253116. doi: 10.1371/journal.pone.0253116 (PMC8270409; doi:10.1371/journal.pone.0253116)
Supplement: Attachment — Submitted filename: PLOS R+R response to reviewers.docx [file pone.0253116.s002.docx]

PLOS reviews

Dear Editors and Reviewers,

We thank the reviewers for their careful responses and reading of the paper. Since we originally wrote and submitted the paper in early summer 2020, there have been numerous changes in the pandemic, as well as the associated literature. We have therefore sought to update our original submission in line with the reviewers’ comments, the evolving research on COVID-19, and the changes in the pandemic itself.

Our primary shift is to break the analysis of the relationship between NPIs and the disease into distinct ‘waves.’ While no standard definition of ‘wave’ is agreed on in the literature, we believe we have identified a workable operationalization that facilitates analysis of the effect of NPIs. We have therefore removed much of the descriptive findings we had previously included, and instead focus on our main regression results. We are grateful to build on numerous similar studies that have sought to estimate the effects of NPIs, and align with them methodologically, while also seeking to add to the literature with a novel approach to periodization of phases of the pandemic in each country.

In addition to this change, the passage of time has allowed us to further expand, analyze, and publish additional details regarding our data on government responses, around which a number of the reviewers’ comments focus. We seek to respond to these specific questions below, while also noting that many additional details have been published in: Hale T, Angrist N, Goldszmidt R, et al. A global panel database of pandemic policies (Oxford COVID-19 Government Response Tracker). *Nat. Hum. Behav.* Published online March 8, 2021. doi:10.1038/s41562-021-01079-8.

Sincerely,

The Authors

| Reviewer | Comments | Response |
| --- | --- | --- |
| R1.1 | 2. Methods, Data Collection: The section needed more details. There were a lot of words without details. The reproducibility might be low. | We have substantially revised this section and included full details on 1) data collection and data sources; 2) Methodology for categorizing waves; 3) Regression analysis; 4) Robustness checks. |
| R1.2 | 3. Methods, Data Collection, “data were collected by . . . ”: I suggest describing the data source in more details to ensure reproducibility. | We have expanded this section. We have also now published a thorough description of the government responses data, which we cite here: Hale T, Angrist N, Goldszmidt R, et al. A global panel database of pandemic policies (Oxford COVID-19 Government Response Tracker). *Nat. Hum. Behav.* Published online March 8, 2021. doi:10.1038/s41562-021-01079-8 |
| R1.3 | 4. Methods, Data Collection, “data collectors coded govenment responses . . . ”: For the ordinal scale, did you mean C1 in Table S1? If so, please refer it to Table S1. In addition, please provide more details on the binary scale. | Table S1 describes each indicator and all points on the ordinal scale. The binary “flag” targeted/general refers to whether a policy applies throughout a whole jurisdiction (general) or just a subset of it (targeted). Full details are provided in: Hale T, Angrist N, Goldszmidt R, et al. A global panel database of pandemic policies (Oxford COVID-19 Government Response Tracker). *Nat. Hum. Behav.* Published online March 8, 2021. doi:10.1038/s41562-021-01079-8 |
| R1.4 | 5. Methods, Data Collection, “several indicators were . . . ”: Similarly, please refer “several indicators” to Table S1. | We have specified the indicators that have targeted/general flags in the text. |
| R1.5 | 6. Methods, Data Collection, “the data over over 170 countries . . . ”: Would it be possible to list all countries (exactly how many countries) in the supplementary materials and indicate which countries’ data contributed to which parts of analyses? | We have specified how many countries are part of each column in the regression results, and provide a list of all countries in the supplement. |
| R1.6 | 7. Methods, Measuring the speed and degree of government non-pharmaceutical interventions, “foreach of the nine relevant policy response indicators . . . ”: I believed the relevant policy response indicators were listed in Table S1. If so, please point them out and spend some sentences describing them. | This section of the article has now been removed. |
| R1.7 | 8. Methods, Measuring the speed and degree of government non-pharmaceutical interventions, “most countries in the time period of analysis adopted most NPIs as a package”: I failed to understand this from Figure S1. | We have now had an opportunity to extensively discuss this pattern in a separate article, which we now cite here. Full details are provided in: Hale T, Angrist N, Goldszmidt R, et al. A global panel database of pandemic policies (Oxford COVID-19 Government Response Tracker). *Nat. Hum. Behav.* Published online March 8, 2021. doi:10.1038/s41562-021-01079-8 |
| R1.8 | 9. Methods, Measuring the speed and degree of government non-pharmaceutical interventions: Were the principal component analysis and principal factor analysis suitable for non-normal variables (i.e., ordinal and/or binary)? | We believe Item Response Theory is a superior method for this kind of data. We have demonstrated this alternative approach in a separate paper, which shows that IRT is highly correlated with the simple approach adopted here. Full details are provided in: Hale T, Angrist N, Goldszmidt R, et al. A global panel database of pandemic policies (Oxford COVID-19 Government Response Tracker). *Nat. Hum. Behav.* Published online March 8, 2021. doi:10.1038/s41562-021-01079-8 |
| R1.9 | 10. Methods, Measuring the speed and degree of government non-pharmaceutical interventions, “the average level of stringency from January 1, 2020 to May 27, 2020”: Why the average start January 1, 2020, but not the date when the first case recorded for a country? | This section of the article has now been removed. |
| R1.10 | 11. Methods, Outcome Variables, “we analyze growth in deaths in terms of the daily log difference . . . ”: Was the difference from that of the previous day? What if the difference was negative? | This section of the article has now been removed. |
| R1.12 | 12. Methods, Outcome Variables, “we consider the maximum daily number of new deaths . . . ”: Would the maximum daily number of new deaths be a fair measurement because not all countries recorded the first death on the same date? Wouldn’t the countries recorded the first death on a later date within the study window have a relatively smaller maximum? Should the maximum be measured at a fixed window since the first recorded death? | This section of the article has now been removed. |
| R1.13 | 13. Methods, Statistical Analysis, “we estimate both cross-sectional models ...”: Somewhere in the Methods section, maybe the Data Collection section, I suggest introducing the cross-sectional and time-series study designs and relating the measuring the speed and degree of government non-pharmaceutical interventions and outcomes to these two study designs. | We have removed the cross-sectional analysis. |
| R1.14 | 14. Methods, Statistical Analysis, “we estimate models that use both time and country fixed effects”: Were there any random effects in the longitudinal models? | We do this as a robustness check. Please see Table S2.1. |
| R1.15 | 15. Methods, Statistical Analysis, “we use the difference in logs as the dependent variable ...”: The outcome must be daily cumulative death events, not daily death events. Otherwise, how did you handle negative difference in the log transformation? What specific longitudinal models were used? | We use the natural log of daily deaths as the dependent variable. This means that our regression coefficients in OLS show the effect of a 1 point change in the stringency index on the the average daily difference in logs, which approximates a percentage point change, which facilitates interpreation. We aim to describe this in the methods and results section. |
| R1.16 | 16. Results, Variation in the speed and intensity of government responses, “Figure 1 shows a positive correlation ...”: How did the residuals look like in this OLS regression? Was the OLS regression suitable to examine this relationship? This also applies to Figure 2. | We no longer included this analysis. |
| R1.17 | 17. Results, Variation in the speed and intensity of government responses: Shouldn’t there be a another model for the relationship between the government SI six weeks prior and the max daily new deaths? | We no longer include this analysis. |
| R1.18 | 18. Results, Variation in the speed and intensity of government responses, “Figure 4 explores these relationships . . . ”: What was the rationale to select these six countries? | We no longer include this analysis. However, the countries were simply chosen to demonstrate a range of relationships. |
| R1.19 | 19. Results, Regression Results: Please introduce the cross-country models in the Statistical Analyses section. | We no longer include this analysis. |
| R1.20 | 20. Figures 1–3: (a) Were all 170 countries included in this analysis? If not, please indicate how many countries were in this analysis and describe the reasons why some countries were not included. (b) What was the criteria for the 12 selected countries? (c) Please remind readers the y-axis was depicted in a log scale. | We no longer include this analysis. Our new tables shows the precise number of countries in each regression, outlined in table 1. |
| R1.21 | 21. Table S2: Why were there 24 parameters (response indicators), not 9? | We no longer include this analysis. |
| R2.2 | 1. In the cross-sectional analysis, the authors calculate a Stringency Index that is averaged across the entire study period (Jan-May 2020), which is then used to calculate the two explanatory variables of primary interest: average stringency and speed of response. They then correlate these variables with the two outcome variables of primary interest: average daily growth in the death rate and zenith death rate. There are several concerns here: | We no longer include the cross-sectional analysis. |
| R2.3 | a) In general, I would like to see more analyses teasing apart the general findings about the Stringency Index. Throughout the Results section, the authors write things like "a 10-point difference in SI would be expected to lead, six weeks later, to a daily growth rate in deaths nearly half a percentage point lower", but what changes would a country government need to implement to do that? eg Can they get there by closing schools and workplaces but keeping bars open? | Because the Index does not weight any indicators, all changes across the ordinal scales are essentially fungible. So a 10 point reduction could be achieved in a reduction in one or more indicators. Nor is it guaranteed that all indicators move in this same direction. So a total shift up or down in the index, or even now change, could reflect a mix of individual indicators rising or falling. In practice, however, governments have tended to raise or lower policies as packages. We discuss this phenomenon extensively in a separate paper. Hale T, Angrist N, Goldszmidt R, et al. A global panel database of pandemic policies (Oxford COVID-19 Government Response Tracker). *Nat. Hum. Behav.* Published online March 8, 2021. doi:10.1038/s41562-021-01079-8 |
| R2.4 | b) The explanatory and outcome variables of primary interest, insofar as they compress longitudinal information into a single observation per country, mask considerable heterogeneity within countries, both over time (ie., temporal heterogeneity) as well as geographically (ie., regional heterogeneity). | We no longer include the cross-sectional analysis. |
| R2.5 | c) It is unclear to me how the authors handled distancing measures that were implemented and then rescinded during the study period. For example, a country may have initiated a "lockdown" in March and then rescinded the lockdown in May. Do the authors use a "once on, always on"/"once treated, always treated" approach here? This is unclear to me from reading the Methods and should be clarified. I _think_ what the authors did is they calculated the Stringency Index for each day in each country, and then averaged across the days. So in a toy example with 3 days, if a country had a Stringency Index value of 0 on day 1, 50 on day 2, and 10 on day 3, the average Stringency Index would be calculated as (0+50+10)/3 = 20. In my understanding, if countries "turned on" and "turned off" social distancing measures at different time points during the study period, the Stringency Index would increase and decrease on a daily basis, but the average Stringency Index over the study period would largely reflect the maximum stringency as well as the number of days that social distancing was "turned on". Some difficulties with this specification can be better understood with a toy example. Supposing for example we have a 10-day study period in which Country A has a Stringency Index of 100 on days 1 & 2, then turns off social distancing completely and has a Stringency Index of 0 on days 3-10. The average Stringency Index for Country A through the study period would therefore be (100+100+0+...+0)/10 = 20. An alternative Country B might have limped along with a Stringency Index of 20 on each day throughout the study period, and would have an average Stringency Index of (20+20+...+20)/10 = 20. Under the authors' scoring, both countries would have equivalent values of the primary explanatory variable despite having arguably very different (formal) social distancing responses. Similarly, compressing the longitudinal data to create the cross-sectional outcome variables (average daily growth in the death rate and zenith death rate) also presents difficulties. Both China and Spain, for example, topped out at around 10,000 daily new cases (I don't know what their comparative death rates were)--and would therefore be treated equivalently in terms of the zenith outcome variable--but China has arguably been much more successful at containing their local epidemic compared with Spain, which is currently in a second surge. | We no longer include the cross-sectional analysis. |
| R2.6 | D) For one of the explanatory variables (speed of response), the authors specified this variable as the number of days until the country achieved a Stringency Index of 40. They provide some text to justify this arbitrary threshold, which is appreciated, but I guess I am just not super convinced, partly because it is not even clear to me what a SI=40 means. The authors state that "almost every country reaches at least this threshold at some point"--were there specific components of the Stringency Index (eg., schools, public events) that drove this particular observation? Why not conduct some sensitivity analyses to see whether their findings are sensitive to the threshold choice? Or better yet, why not simply model the slope (ie., "speed of response") directly? | We no longer include this analysis. |
| R2.7 | e) In terms of geographic heterogeneity, the authors did not account for the substantial within-country heterogeneity. The US, for example, would treated as a single country with a single observation for level of stringency and outcome. Obviously this is untrue, both in terms of the implementation of social distancing measures (Siedner et al. PLoS Med 2020;17:e1003244) as well as in the relaxation/rescinding of social distancing measures (Tsai et al. medRxiv 2020 Aug 7, doi:10.1101/2020.07.15.20154534)--and not just in the US, but also in other countries with a much stronger national level response, eg., China (Maier & Brockmann, Science 2020;368:742-6; Lai et al. Nature 2020 May 4, doi:10.1038/s41586-020-2293-x). The authors mention this limitation in the Discussion on page 14, but in my opinion this limitation is so glaring that I am not sure what we can learn from the cross-sectional analyses. (Perhaps the authors can be clearer about this in revised text.) The authors cite Cauchemez et al. (ref #40) in noting the significant country-to-country variability in government stringency and the 2009 influenza pandemic. But this is also well known in the context of COVID in the US (eg., Holtz et al. PNAS 2020;117:19837-43). Certainly if the measurement error (ie., due to lack of accounting for within-country heterogeneity) in the outcome variables is non-differential, the estimated associations would simply bias toward the null. But the same cannot be said for measurement error in the explanatory variables. Other investigators have dealt with this by collecting data on social distancing measures implemented at lower/subnational levels of jursdiction (eg., Hsiang et al. Nature 2020;584:262-7) or focused on a single country (as we did in our own work, Siedner et al. PLoS Med 2020;17:e1003244 and Tsai et al. medRxiv 2020 Aug 7, doi:10.1101/2020.07.15.20154534). | This is a strong critique that we recognize as a limitation of our data structure. Since the original submission, we have sought to address this issue by collecting comprehensive data on sub-national government responses in large, heterogenous countries. This additional data collection allows us to add a parallel analysis for 26 Brazilian states and 71 Brazilian cities, and for 52 US states and territories (DC, Puerto Rico), which we report in Table S2.3 as a robustness check. |
| R2.8 | 2. The authors also conducted longitudinal analyses, which would address concerns about masking within-country temporal heterogeneity (but not concerns about masking within-country geographic heterogeneity). Here they estimate fixed effects regression models, specifying country i on day t as the unit of analysis, lagging the explanatory variable by 6 weeks. | NA |
| R2.9 | a) The fixed effects estimates are driven entirely by within-country changes in the Stringency Index and by within-country changes in mortality. So I would like to see some descriptive statistics (eg., intraclass correlations etc) quantifying the extent of within vs. between country variation. | During the period of analysis, the Stringency Index shifts significantly in every country, from 0 to, in some cases, 100. Similarly, countries have seen enormous variation in deaths over the course of the pandemic. |
| R2.10 | b) It is unclear to me why the authors chose a lag of 6 weeks. In my opinion, there is considerable uncertainty about the appropriate interval one would expect between implementation of social distancing measures and any response in mortality. While the median incubation period is likely on the order of 3-5 days, the median time between symptom onset and death varies widely (eg., 8 days in Italy, two weeks in China). (Death probabilities, and time to death, would also be highly contingent on local comorbidity epidemiology as well as health system factors, but these would be unlikely to vary during the short time frame of the study and would therefore be differenced out in the analysis.) In our own work, we took a much more exploratory approach to modeling the association between social distancing and mortality (Siedner et al. PLoS Med 2020;17:e1003244). Perhaps the authors might adopt a similar approach and specify different lag periods to probe the extent to which their findings are sensitive to the choice of lag. | Based on our reading of the evolving literature, we have updated this to 28 days. We discuss this choice in the article in the methods section. |
| R2.11 | c) The authors motivate their lagged analysis, at the bottom of page 7, by suggesting that reverse causality could be at play. To rule this out, they probably should just probe the data rather than speculate. One could imagine a companion analysis in which the authors estimate the association between lagged deaths and the Stringency Index (to see whether it is indeed the case that "deaths might trigger a policy response"). As above, the extent to which the forward/reverse associations differ would depend on the extent of within-country variation over time. | We believe our periodization of countries by waves helps to address this concern. |
| R2.12 | d) It would be helpful if the authors could write out the estimating equations. It was not clear to me, either from the text or in Table 1, whether the authors estimated a single regression model containing both of the explanatory variables of interest (and also adjusting for the covariates listed in footnotes b & c), or whether the authors estimated two regression models containing 1 of the explanatory variables of interest (each of which adjusted for the covariates listed in footnotes b & c). | We estimate a single regression model in each column. |
| R2.13 | 3. The authors justify their use of death rates (rather than case rates) by asserting that they are likely to be reported more consistently. Is this true? (eg., Weinberger et al. JAMA Intern Med 2020 Jul 1, doi:10.1001/jamainternmed.2020.3391; Rivera et al. medRxiv 2020 Jun 27, doi:10.1101/2020.05.04.20090324). | We have followed the evolving literature on this debate and have updated the citations to reflect this. In our reading the evidence points now even more strongly to deaths as the “least bad” indicator of disease prevalence. |
| R2.14 | 4. Table S1-- Could the authors perhaps be a little more concrete about what goes into the different coding elements? For example, are restaurants included in C2 (workplaces) or C4 (restrictions on gatherings)? Are churches included in C3 (public events) or C4 (gatherings)? | We have now had the opportunity to discuss and describe the data extensively in a separate publication. Hale T, Angrist N, Goldszmidt R, et al. A global panel database of pandemic policies (Oxford COVID-19 Government Response Tracker). *Nat. Hum. Behav.* Published online March 8, 2021. doi:10.1038/s41562-021-01079-8 |
| R2.15 | 5. To calculate the composite Stringency Index, the authors simply added up the individual components listed in Table S1. Obviously there are problems with granting equal weights to the different components, eg. canceling all public events (2 points) vs. closing some schools (also 2 points). This should be mentioned as a limitation.  b) To avoid imposing equal weights on the different elements, why not extract the first principal component (Table S2) and use that as the Stringency Index? Such an approach would allow the data to drive the weighting, similar to Filmer & Pritchett (Demography 2001;38:115-132). | We address these alternatives in the discussion section. We believe there are pros and cons to any approach, which we have discussed in some detail in Hale T, Angrist N, Goldszmidt R, et al. A global panel database of pandemic policies (Oxford COVID-19 Government Response Tracker). *Nat. Hum. Behav.* Published online March 8, 2021. doi:10.1038/s41562-021-01079-8. On further reflection, do not think principal component analysis, which we had originally included, is appropriate for the ordinal data structure. Instead, an Item Response Theory approach would be preferred. However, as we demonstrate in the paper cited above, and IRT index is highly correlated with the approach we use here, which we believe has the additional merit of being transparent and easy to interpret. |
| R2.16 | 6. While the Discussion section refers vaguely to "potential confounders", there is no mention of spontaneous behavioral change that may have occurred prior to implementation of any distancing measures. Such behavioral change was highly conditioned, both at the individual level (eg, Papageorge et al. COVID Econ 2020;40:1-45; Weill et al PNAS 2020 Aug 18;117(33):19658-19660) and at the area level (eg., Wright et al. SSRN 17 Jun 2020, doi:10.2139/ssrn.3573637), by preexisting sociodemographic factors, which is important from an equity perspective; and this evidence of pre-policy behavioral change also reinforces the notion that the epidemic itself, rather than lockdown-style distancing measures, was the cause of any observed economic fallout (eg., Sheridan et al. PNAS 2020 Aug 25;117 (34):20468-20473). To the extent that implementation of any distancing measures may have been implemented in response to either a worsening local epidemic or behavior change already occurring, the authors estimates are likely to be biased away from the null rather than toward the null--which would lead them to overestimate the effectiveness of social distancing measures in curbing the COVID epidemic. | We agree and have noted this limitation in the Discussion. |
| R2.17 | 7. There are several priority statements scattered throughout the manuscript (eg, page 3, "most comprehensive view"; page 15, "most comprehensive test"; etc). I am uncertain as to whether this is truly the case. For example, Islam et al. (BMJ 2020;370:m2743-- not cited in this manuscript, which is probably an oversight) used the authors' data on distancing measures to estimate the association between distancing measures and COVID-19 incidence-- this does not necessarily undercut the novelty of the database on distancing measures (although cf. Cheng et al. Nature Hum Behav 2020;4:756-768; Zheng et al. Scientific Data 2020;7:286; and Desvars-Larrive et al. Scientific Data 2020;7:285-- I am sure there are others), but it does tend to undercut the novelty of the analyses presented in this specific manuscript. In any case, the manuscript would probably be improved with (a) more comprehensive referencing of these alternative efforts; (b) better contextualizing of the present analyses in relation to other published literature and preprints (how does the authors' database compare with these other efforts? what are the gaps in the alternative databases and how do they compare with the authors' database? etc); and (c) deleting the priority claims, which are probably unnecessary anyway. | We have removed this language. |
